# Serum A-FABP Is Increased and Closely Associated with Elevated NT-proBNP Levels in Type 2 Diabetic Patients Treated with Rosiglitazone

**DOI:** 10.1371/journal.pone.0027032

**Published:** 2011-10-28

**Authors:** Mi Zhou, Yuqian Bao, Junxi Lu, Jian Zhou, Weiping Jia

**Affiliations:** 1 Department of Medicine, Shanghai Jiao Tong University School of Medicine, Shanghai, China; 2 Department of Endocrinology and Metabolism, Shanghai Jiao Tong University Affiliated Sixth People's Hospital, Shanghai Diabetes Institute, Shanghai Key Laboratory of Diabetes Mellitus, Shanghai Clinical Center for Diabetes, Shanghai, China; University of Hong Kong, China

## Abstract

**Background:**

Adipocyte fatty acid-binding protein (A-FABP) has been shown to play important roles in the development of metabolic syndrome, diabetes, and cardiovascular diseases. In this study we investigated the possible role of A-FABP in the development of cardiac dysfunction related to rosiglitazone treatment.

**Methodology/Principal Findings:**

A total of 84 patients with newly diagnosed type 2 diabetes were treated with rosiglitazone for 48 weeks. Circulating A-FABP and N-terminal pro-brain natriuretic peptide (NT-proBNP) levels were determined at baseline and repeated at 24 and 48 weeks. After the 48-week rosiglitazone treatment period, serum levels of both A-FABP and NT-proBNP increased progressively and significantly (*P*<0.01). Serum levels of A-FABP were demonstrated to be positively correlated with gender and waist circumference both at baseline and the end of the study, and with age, body mass index (BMI), total cholesterol (TC), triglyceride (TG), low-density lipoprotein cholesterol (LDL-C), and NT-proBNP at 48 weeks (all *P*<0.05). In addition, changes in A-FABP were significantly and positively correlated with changes in NT-proBNP (r = 0.239, *P* = 0.039). Furthermore, multiple stepwise regression analysis showed that the changes in A-FABP were independently and positively associated with changes in NT-proBNP after adjusting for confounding factors (*β* = 0.320, *P* = 0.007).

**Conclusions/Significance:**

Rosiglitazone-mediated increase of A-FABP is closely associated with the elevation of NT-proBNP, a well-established marker of cardiac dysfunction. The findings of our study imply that A-FABP may mediate the cross-talk between heart and adipose tissue.

## Introduction

Adipocyte fatty acid-binding protein (A-FABP), also known as FABP4, is a cytosolic lipid chaperone that reversibly binds with high affinity to hydrophobic ligands, such as saturated and unsaturated long-chain fatty acids [1]. It is predominantly expressed in mature adipocytes and activated macrophages [2], [3]. Emerging evidences suggest that A-FABP plays an important role in lipid-mediated biological processes, and is closely associated with obesity, diabetes, the metabolic syndrome, and the development of atherosclerosis [4]–[7]. Recently, the relationship between A-FABP and cardiovascular disease has raised much attention. A genetic variant associated with increased A-FABP mRNA expression in adipose tissue has been reported to predict coronary artery disease in homozygous subjects [8]. Several studies found the relationship between serum A-FABP levels and coronary atherosclerosis [9], [10]. We have recently demonstrated that A-FABP is closely associated with the presence and severity of coronary artery disease [11].

Thiazolidinediones (TZDs) are peroxisome proliferator activated receptor γ (PPARγ) agonists that specifically augment insulin sensitivity in peripheral tissues. The cardiovascular adverse events of rosiglitazone such as fluid retention and risk of ischaemic cardiovascular events and heart failure during the treatment of diabetes have been recognized [12], [13]. The precise biological mechanism responsible for the cardiovascular risk of rosiglitazone remains uncertain. Cell-based assay has demonstrated that the expression of A-FABP can be induced by PPARγ agonists [14], which can also increase the circulating levels of brain natriuretic peptide (BNP) [15]. Circulating concentrations of N-terminal pro-brain natriuretic peptide (NT-proBNP) has been proposed as a biomarker of cardiovascular disease and in particular a marker of heart failure, and holds promise as a tool to screen the general population both for prevalence of underlying cardiac structural and functional abnormalities as well as for the future development of cardiovascular events [16], [17]. Although the adverse effect of rosiglitazone on fluid dynamics has been proposed as a probable mechanism of its greater risk of congestive heart failure, it is unclear whether A-FABP is also involved in the PPARγ agonists-induced changes of cardiovascular function. In the present study, we investigated the long-term effects of rosiglitazone on circulating A-FABP and NT-proBNP levels in patients with newly diagnosed type 2 diabetes (T2DM), and the possible role of A-FABP in the development of cardiac dysfunction.

## Methods

### Ethics Statement

All subjects gave written informed consent, and the study was approved by the ethics committee of Shanghai Jiao Tong University Affiliated Sixth People's Hospital and complied with the Declaration of Helsinki.

### Participants

Subjects with newly diagnosed T2DM were recruited from the outpatient clinics of 10 academic hospitals in Shanghai. The diagnosis of T2DM was based on 1999 World Health Organization criteria [18]. The criteria for study inclusion and exclusion have been described previously [19], [20]. This study includes data from 84 patients (60 men, 24 women) aged 52.6±8.9 years. All patients were treated with rosiglitazone (Avandia; GlaxoSmithKline, Munich, Germany) for 48 weeks which were comprised of 8 visits. Fasting plasma glucose (FPG) and 2-h postchallenge plasma glucose (2hPG) were monitored at each visit. The initial dose of rosiglitazone was 4 mg/d and escalated to 8 mg/d in patients who failed to attain FPG <7 mmol/l and/or 2hPG <11 mmol/l. Liver function tests were performed every 12 weeks. Lipid profile and arginine stimulation test which was to estimate acute insulin secretion of islet β cells were performed at baseline and repeated at 24 and 48 weeks.

### Anthropometric evaluation

A complete physical examination including height, weight, waist circumferences, and systolic blood pressure (SBP) and diastolic blood pressure (DBP) were performed on each subject at baseline, 24 weeks and 48 weeks after the initiation of rosiglitazone therapy. Body mass index (BMI) was calculated as weight in kilograms divided by squared height in meters (kg/m^2^). Waist circumference was measured midway between the lowest rib and the superior border of iliac crest on midaxillary line.

### Biochemical measurements

Blood samples were collected after an overnight fast. Arginine stimulation tests were performed on all the patients. At time 0, arginine hydrochloride (10% arginine hydrochloride of 50 ml, 5 g) was injected intravenously within 30–60 s. Blood samples were drawn at times 0, 2, 4, and 6 min after injection for glucose and insulin measurements [21]. Plasma glucose concentrations were measured using glucose oxidase method. Serum insulin was assayed via radioimmunoassay (Linco Research, St Charles, MO, USA). Glycated hemoglobin A1c (HbA1c) values were determined by high performance liquid chromatography (Bio-Rad Laboratories, Hercules, CA, USA). Serum lipid profiles, including total cholesterol (TC), triglyceride (TG), high-density lipoprotein cholesterol (HDL-C), and low-density lipoprotein cholesterol (LDL-C) were measured by enzymatic procedures using an autoanalyser (Hitachi 7600–020; Hitachi, Tokyo, Japan). NT-proBNP was assayed by chemiluminescence (Cobas 6000, Roche Diagnostics GmbH, Mannheim, Germany). A-FABP levels were assayed by sandwich ELISA (Antibody and Immunoassay Services, the University of Hong Kong). The intra- and inter assay variations were 4.5 and 6.7%, respectively.

### Evaluation of insulin resistance (IR) and β-cell function

IR and basal insulin secretion were estimated using the homeostasis model assessment (HOMA) index based on fasting glucose and insulin measurements as follows: HOMA-IR =  fasting insulin (mU/l)×FPG (mmol/l)/22.5. HOMA of beta cell function (HOMA-B%) = 20×fasting insulin/(FPG-3.5) [22]. The acute insulin response (AIR) was used to examine the amount of acute phase insulin secretion after arginine stimulation, and was calculated as the mean insulin value of 2, 4, and 6 min minus fasting insulin concentration.

### Statistical analysis

All analyses were performed using SPSS version 13.0 (SPSS, Chicago, IL). Data were expressed as means ± SD for normal distributions or median (interquartile range) for skewed variables. Data that were not normally distributed as determined by the Kolmogorov-Smirnov test were logarithmically transformed before analysis. To test the effect of rosiglitazone treatment during the examination period, we used repeated-measures ANOVA to compare data from baseline and weeks 24 and 48, and multiple comparisons were performed applying the Bonferroni correction. Pearson's correlation was used to evaluate the relationships among continuous variables. Multiple stepwise regression analysis was performed to determine the independent parameters correlated with A-FABP, NT-proBNP, and changes in A-FABP and NT-proBNP. *P* < 0.05 was considered significant.

## Results

### Clinical characteristics of the patients

The demographic and biochemical characteristics of the study subjects at baseline and after 24 and 48 weeks treatment with rosiglitazone were summarized in Table 1. Compared to baseline, DBP, FPG, 2hPG, HbA1c, and HOMA-IR all decreased significantly at week 24 and 48, whereas HDL-c increased significantly at week 48 (all *P*<0.01).

**Table 1:** Clinical characteristics of study subjects.

	Baseline	24 weeks	48 weeks
Waist circumference (cm)	88.2±7.3	87.1±9.0	87.4±8.3
BMI (kg/m^2^)	25.0±2.9	24.8±3.2	24.8±3.0
SBP (mmHg)	128.1±17.3	127.7±16.7	125.7±17.1
DBP (mmHg)	83.4±10.0	80.7±9.3*	79.4±9.1*
TC (mmol/l)	5.4±1.2	5.5±1.2	5.5±1.2
TG (mmol/l)†	1.9 (1.2–2.8)	1.8 (1.3–2.5)	1.9 (1.1–2.8)
HDL-c (mmol/l)	1.2±0.2	1.3±0.3	1.3±0.3*
LDL-c (mmol/l)	3.4±1.0	3.2±0.9	3.2±0.9
Fasting insulin (mU/l)	14.2±7.1	15.8±7.5	16.2±9.8
FPG (mmol/l)	8.9±1.8	6.3±0.9*	6.5±1.2*
2hPG (mmol/l)	13.5±3.2	8.3±2.5*	8.7±2.1*
HbA1c (%)	8.2±1.5	6.3±0.6*	6.3±0.7*
HOMA-IR	6.5±3.7	4.1±2.4*	4.5±3.4*
HOMA-B%†	72.2 (44.3–123.3)	94.2 (68.3–152.8)	91.6 (60.7–134.3)
AIR (mU/l)†	22.2 (12.5–36.3)	19.0 (11.1–34.6)	22.7 (11.3–39.0)

Data are mean ± SD or median (interquartile range). AIR: acute insulin response; BMI: body mass index; DBP, diastolic blood pressure; FPG: fasting plasma glucose; 2hPG: 2-h postchallenge plasma glucose; HbA1c: glycated hemoglobin A1c; HDL-c: high density lipoprotein cholesterol; HOMA-IR: homeostasis model assessment index of insulin resistance; HOMA-B%: homeostasis model assessment index of beta cell function; LDL-c: low density lipoprotein cholesterol; SBP, systolic blood pressure; TC: total cholesterol; TG: triglyceride.

† Log transformed before analysis.

*P<0.01 vs. baseline.

**Figure 1:**
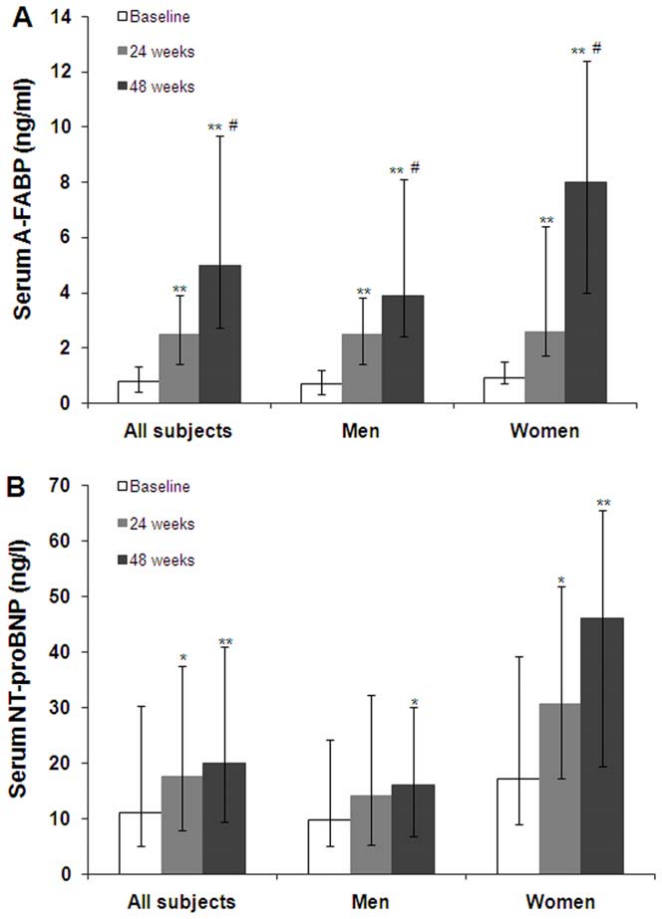
Serum A-FABP and NT-proBNP concentrtions at baseline and after 24 and 48 weeks of rosiglitazone therapy in 84 patients with newly diagnosed T2DM. Data are median with interquartile range. A: Serum A-FABP levels at baseline and after 24 and 48 weeks of rosiglitazone therapy were 0.8 (0.4–1.3) ng/ml, 2.5 (1.4–3.9) ng/ml, and 5.0 (2.7–9.7) ng/ml for all patients, 0.7 (0.3–1.2) ng/ml, 2.5 (1.4–3.8) ng/ml, and 3.9 (2.4–8.1) ng/ml for men, and 0.9 (0.7–1.5) ng/ml, 2.6 (1.7–6.4) ng/ml, and 8.0 (4.0–12.4) ng/ml for women. B: Serum NT-proBNP levels at baseline and after 24 and 48 weeks of rosiglitazone therapy were 11.1 (5.0–30.3) ng/l, 17.6 (7.8–37.6) ng/l, and 20.0 (9.4–40.9) ng/l for all subjects, 9.7 (5.0–24.2) ng/l, 14.2 (5.3–32.3) ng/l, and 16.0 (6.7–30.1) ng/l for men, and 17.2 (9.0–39.2) ng/l, 30.7 (17.3–51.9) ng/l, and 46.1 (19.4–65.7) ng/l for women. * *P*<0.01 versus baseline. ** *P*<0.001 versus baseline. # *P*<0.001 versus 24 weeks.

Serum levels of A-FABP and NT-proBNP are higher in women than that in men at both baseline (both *P*<0.01) and the end of the study (both *P*<0.05). As shown in Figure 1, 24 and 48 weeks after the treatment with rosiglitazone, serum levels of A-FABP increased progressively and significantly in both genders (all *P*<0.001). As compared with week 24, A-FABP levels also significantly increased at the study end (*P*<0.001). Serum NT-proBNP demonstrated similar increasing trend, with significant higher levels at 48 weeks for men and at both 24 and 48weeks for women compared to baseline (all *P*<0.01).

### Correlation analysis and multiple linear regression analysis showing parameters related to A-FABP and NT-proBNP

At baseline (Table 2), univariate analysis revealed significant correlations of serum A-FABP with gender (women) and waist circumference (both *P*<0.05). Serum NT-proBNP was positively correlated with age and gender (women) (both *P*<0.05), and negatively correlated with HbA1c, HOMA-IR, and AIR (all *P*<0.05). On multiple stepwise regression analysis, gender (*P* = 0.048) and BMI (*P* = 0.020) were independently related to A-FABP, and age (*P*<0.001), LDL-c (*P* = 0.032), and HOMA-IR (*P* = 0.019) were independently correlated with NT-proBNP.

**Table 2:** Anthropometric and biochemical parameters showing significant correlations with serum A-FABP and NT-proBNP at baseline.

	A-FABP				NT-proBNP			
	Univariate†		Multivariate*		Univariate†		Multivariate#	
	*r*	*P*	*β*	*P*	*r*	*P*	*β*	*P*
Age	0.033	0.769			0.554	<0.001	0.421	<0.001
Gender (women)	0.208	0.034	0.207	0.048	0.249	0.022		
Waist circumference	0.203	0.039			0.038	0.729		
BMI	0.205	0.062	0.345	0.020	-0.089	0.423		
SBP	0.038	0.731			0.171	0.075		
DBP	0.056	0.610			0.033	0.731		
TC	-0.028	0.803			-0.137	0.156		
TG	0.134	0.176			-0.181	0.059		
HDL-c	-0.116	0.241			0.211	0.054		
LDL-c	0.175	0.076			-0.166	0.085	-0.230	0.032
FPG	-0.069	0.535			-0.195	0.078		
2hPG	0.142	0.207			-0.113	0.317		
Fasting insulin	-0.085	0.440			-0.028	0.773		
HbA1c	-0.139	0.161			-0.236	0.030		
HOMA-IR	0.178	0.123			-0.277	0.016	-0.252	0.019
HOMA-B%	0.045	0.672			0.061	0.599		
AIR	0.089	0.367			-0.215	0.025		
A-FABP					0.112	0.258		

*β*, Standardized regression coefficients.

† Pearson correlation analyses were performed.

*A multiple stepwise regression analysis was performed. Variables included in the original model are age, gender, waist circumference, BMI, and LDL-c.

# A multiple stepwise regression analysis was performed. Variables included in the original model are age, gender, SBP, TG, HDL-c, LDL-c, FPG, HbA1c, HOMA-IR, and AIR.

After 48 weeks of rosiglitazone therapy (Table 3), serum levels of A-FABP were demonstrated to be positively correlated with age, gender, waist circumference, BMI, TC, TG, LDL-c, and NT-proBNP (all *P*<0.05). Serum NT-proBNP was shown to be positively correlated with age, gender, SBP, HDL-c, and A-FABP, and negatively correlated with BMI, FPG, fasting insulin, HOMA-IR, and AIR (all *P*<0.05). On multiple stepwise regression analysis, age (*P* = 0.002), gender (*P* = 0.009), BMI (*P* = 0.023), TG (*P* = 0.019), and LDL-c (*P* = 0.002) were independently and positively related to A-FABP, whereas age (*P*<0.001), gender (*P* = 0.036), and HOMA-IR (*P* = 0.026) were independent factors for NT-proBNP. However, the relationship between A-FABP and NT-proBNP was abolished after multi-adjustment.

**Table 3:** Anthropometric and biochemical parameters showing significant correlations with serum A-FABP and NT-proBNP after the 48-week rosiglitazone treatment.

	A-FABP				NT-proBNP			
	Univariate†		Multivariate*		Univariate†		Multivariate#	
	*r*	*P*	*β*	*P*	*r*	*P*	*β*	*P*
Age	0.376	<0.001	0.312	0.002	0.587	<0.001	0.492	<0.001
Gender (women)	0.285	0.009	0.243	0.009	0.355	0.001	0.200	0.036
Waist circumference	0.217	0.049			-0.127	0.259		
BMI	0.231	0.035	0.229	0.023	-0.231	0.038		
SBP	0.171	0.125			0.227	0.043		
DBP	0.161	0.147			-0.084	0.461		
TC	0.480	<0.001			0.014	0.897		
TG	0.351	0.001	0.241	0.019	-0.100	0.369		
HDL-c	-0.068	0.539			0.281	0.011		
LDL-c	0.451	<0.001	0.303	0.002	0.021	0.850		
FPG	0.051	0.663			-0.237	0.035		
2hPG	-0.181	0.107			-0.059	0.603		
Fasting insulin	0.148	0.179			-0.394	<0.001		
HbA1c	0.077	0.493			-0.019	0.868		
HOMA-IR	0.003	0.981			-0.426	<0.001	-0.218	0.026
HOMA-B%	0.031	0.790			-0.137	0.252		
AIR	0.034	0.757			-0.275	0.013		
A-FABP					0.250	0.024		

*β*, Standardized regression coefficients.

† Pearson correlation analyses were performed.

*A multiple stepwise regression analysis was performed. Variables included in the original model are age, gender, waist circumference, BMI, TC, TG, LDL-c, and NT-proBNP.

# A multiple stepwise regression analysis was performed. Variables included in the original model are age, gender, BMI, SBP, HDL-c, FPG, fasting insulin, HOMA-IR, AIR, and A-FABP.

### Relationship between changes in A-FABP and NT-proBNP versus changes in anthropometric and biochemical parameters

As shown in Table 4, during the 48-week treatment period, changes in A-FABP were negatively associated with changes in HDL-c, and positively correlated with changes in waist circumference and TG (all *P*<0.05). Changes in serum NT-proBNP were negatively correlated with changes in fasting insulin (*P* = 0.003). Furthermore, changes in A-FABP were significantly and positively correlated with changes in NT-proBNP (*P* = 0.039). Multiple stepwise regression analysis further showed that changes in NT-proBNP and changes in A-FABP were independently and positively associated with each other after adjusting for confounding factors (*β* = 0.320, *P* = 0.007). In addition, changes in A-FABP and NT-proBNP were independently associated with changes in waist circumference and BMI, respectively (both *P*<0.05).

**Table 4:** Changes in anthropometric and biochemical parameters showing significant correlations with changes in serum A-FABP and NT-proBNP during the 48-week rosiglitazone treatment.

	Δ A-FABP				Δ NT-proBNP			
	Univariate†		Multivariate*		Univariate†		Multivariate#	
	*r*	*P*	*β*	*P*	*r*	*P*	*β*	*P*
Age	0.372	0.001	0.289	0.014	0.403	<0.001	0.240	0.044
Gender (women)	0.237	0.030			0.290	0.012	0.243	0.024
Δ Waist circumference	0.242	0.028	0.309	0.004	-0.099	0.403		
Δ BMI	0.081	0.468			-0.208	0.076	-0.215	0.048
ΔSBP	-0.169	0.129			-0.127	0.284		
ΔDBP	-0.143	0.200			-0.106	0.371		
ΔTC	0.120	0.275			-0.173	0.138		
Δ TG	0.217	0.047			-0.119	0.309		
Δ HDL-c	-0.290	0.007			-0.011	0.927		
Δ LDL-c	0.045	0.685			-0.151	0.197		
Δ FPG	0.108	0.358			-0.166	0.167		
Δ 2hPG	-0.073	0.528			-0.112	0.354		
Δ Fasting insulin	-0.032	0.776			-0.333	0.003		
Δ HbA1c	0.069	0.543			0.041	0.735		
Δ HOMA-IR	0.017	0.885			-0.180	0.148		
Δ HOMA-B%	-0.061	0.604			-0.221	0.074		
Δ AIR	0.097	0.381			-0.079	0.501		
Δ NT-proBNP	0.239	0.039	0.289	0.014				
Δ A-FABP					0.239	0.039	0.320	0.007

Δ, differences between after and before treatment.

*β*, Standardized regression coefficients.

† Pearson correlation analyses were performed.

*A multiple stepwise regression analysis was performed. Variables included in the original model are age, gender, Δ waist circumference, Δ TG, Δ HDL-c, and Δ NT-proBNP.

# A multiple stepwise regression analysis was performed. Variables included in the original model are age, gender, Δ BMI, Δ fasting insulin, Δ HOMA-B%, and Δ A-FABP.

## Discussion

In the present study, we have shown that serum levels of both A-FABP and NT-proBNP were increased progressively and significantly after the treatment with rosiglitazone in a cohort of newly diagnosed type 2 diabetic patients, and have demonstrated for the first time that changes in serum levels of A-FABP were independently and positively associated with changes in NT-proBNP.

*In vitro* studies suggest that A-FABP is regulated by PPARγ agonists such as rosiglitazone not only in human adipocytes but also in macrophages [14], [23]. A-FABP expression is activated by PPARγ-signaling through the PPRE element present in the A-FABP gene promoter [24]. Rosiglitazone treatment not only caused an increase in A-FABP mRNA and protein levels in human adipocytes but also in A-FABP secretion from adipocytes [25]. However, few clinical studies have reported the effect of rosiglitazone on serum levels of A-FABP. In this study, in accordance with these cell-based observations, we found serum levels of A-FABP increased progressively with rosiglitazone therapy in diabetic patients. An approximately 6-fold increase in serum A-FABP levels was observed after the 48-week treatment. Likewise, a cross-sectional study demonstrated that serum A-FABP levels were significantly higher in TZD-treated type 2 diabetic patients than that in control subjects. In addition, they also reported in their subgroup analysis on 6 patients that another PPARr agonist pioglitazone increased serum A-FABP levels significantly after a 12-week treatment [25]. Therefore, our study provides the clinical evidence that rosiglitazone act as an important regulator in A-FABP production in humans.

Another novel observation of this study is the independent and positive relationship between changes in serum levels of A-FABP and NT-proBNP. It has been reported previously that plasma BNP levels are increased with impaired ventricular function in the treatment of diabetic patients with TZD [15], [26]. In the present study we found a significant increase in serum NT-proBNP levels following rosiglitazone therapy. The precise mechanisms responsible for the increase in NT-proBNP levels associated with rosiglitazone treatment remain unclear. One probable explanation is the result of increased cardiac wall stretch caused by volume retention which is considered as the main adverse event of TZD therapy. Christos Sambanis *et al.* reported that pioglitazone does not alter echocardiographic parameters even though it increases NT-proBNP after a 3 month treatment period in 44 patients with T2DM, and speculated that it may represent a reaction to volume overload [27]. It has been suggested that serial monitoring changes in BNP levels over time is beneficial to potentially enable the detection of volume overload secondary to TZD use and the onset of cardiac dysfunction before echocardiographic changes [28]. Although NT-proBNP levels of the patients during the process of this study are within normal ranges, we could not exclude a potent fluid retention, which may be developed as a result of rosiglitazone therapy. In addition, A-FABP may promote the increased secretion of NT-proBNP through its direct actions on cardiomyocytes. Notably, a recent study suggested that A-FABP secreted by human adipocytes in extracellular medium was able to directly suppress cardiomyocyte contraction by attenuating intracellular Ca^2+^ levels [29]. Our finding on the independent association between changes in serum A-FABP and NT-proBNP suggested an interaction may exist between adipose tissue and heart. We therefore speculate that the elevated circulating levels of A-FABP released from subcutaneous and/or visceral adipose tissue in response to rosiglitazone therapy may play an adverse role on cardiomyocyte function, causing an increased synthesis of BNP. Furthermore, epicardial adipose tissue has been shown to express A-FABP and is related to the components of the metabolic syndrome [30]. As there is no fibrous fascial layer between epicardial adipose tissue and the underlying myocardium, A-FABP derived from epicardial adipose tissue may also influence the expression and secretion of BNP through direct paracrine effect. Besides that, A-FABP may exert adverse impact on cardiac function through aggravating the pro-inflammatory responses [31]. However, the precise mechanism underlying the independent association between A-FABP and NT-proBNP requires further investigation.

Consistent with previous studies [4], [5], serum levels of A-FABP in our type 2 diabetic patients were independently associated with lipid and parameters of adiposity such as TG, LDL-c and BMI, especially after the 48-week rosiglitazone treatment. An independent relationship between changes in serum A-FABP and changes in waist circumference was also observed. It is known that A-FABP binds fatty acids with high affinity and mediates intracellular lipid trafficking [32]. Therefore, our findings support the role of A-FABP as a key player of obesity-related metabolic disorders. Interestingly, our study also demonstrated an independent and negative relationship of serum NT-proBNP with HOMA-IR both at baseline and the end of the study. Recently, several studies reported the link between obesity and natriuretic peptide levels, demonstrating an inverse relationship of BMI with BNP and NT-proBNP concentration in subjects with and without heart failure [33], [34]. Multiple mechanisms may contribute to these inverse associations. On the one hand, impaired synthesis and diminished release of NT-proBNP from the heart with increasing BMI are likely to play a role [34]. On the other hand, natriuretic peptides also exert impact on fat cells. BNP was found to be able to stimulate lipolysis in human fat cells through a cGMP-dependent protein kinase signaling pathway [35]. Therefore, the negative relationship between NT-proBNP and cardiovascular risk factors in our study also suggests a reciprocal regulation between heart and fat metabolism.

This study is limited by the lacking of data on echocardiography. Therefore, we could not provide solid evidence on the change of cardiac structure and systolic or diastolic function, as well as their relationship with the increment of serum A-FABP. Further prospective studies including echocardiographic evidence are warranted to clarify whether the association between A-FABP and NT-proBNP following rosiglitazone therapy is dependent on the cardiovascular function.

In summary, our study provide the first evidence that changes in serum levels of A-FABP are independently associated with changes in serum NT-proBNP following rosiglitazone treatment. The findings of our study suggest A-FABP may play an important role in early cardiac dysfunction and imply that A-FABP may mediate the cross talk between the heart and adipose tissue.
